# The Effect of the Thickness of the Sensitive Layer on the Performance of the Accumulating NO_x_ Sensor

**DOI:** 10.3390/s120912329

**Published:** 2012-09-10

**Authors:** Andrea Groß, Miriam Richter, David J. Kubinski, Jacobus H. Visser, Ralf Moos

**Affiliations:** 1 University of Bayreuth, Zentrum für Energietechnik, Lehrstuhl für Funktionsmaterialien, Bayreuth 95440, Germany; 2 Ford Research and Advanced Engineering, Dearborn, MI 48124, USA

**Keywords:** lean NO_x_ trap (LNT), NO_x_ storage and reduction catalyst (NSR), low ppm-level NO_x_ sensing, semiconducting gas sensor, linear measurement range adaption, carbonate nitrate conversion

## Abstract

A novel and promising method to measure low levels of NO_x_ utilizes the accumulating sensor principle. During an integration cycle, incoming NO_x_ molecules are stored in a sensitive layer based on an automotive lean NO_x_ trap (LNT) material that changes its electrical resistivity proportional to the amount of stored NO_x_, making the sensor suitable for long-term detection of low levels of NO_x_. In this study, the influence of the thickness of the sensitive layer, prepared by multiple screen-printing, is investigated. All samples show good accumulating sensing properties for both NO and NO_2_. In accordance to a simplified model, the base resistance of the sensitive layer and the sensitivity to NO_x_ decrease with increasing thickness. Contrarily, the sensor response time increases. The linear measurement range of all samples ends at a sensor response of about 30% resulting in an increase of the linearly detectable amount with the thickness. Hence, the variation of the thickness of the sensitive layer is a powerful tool to adapt the linear measurement range (proportional to the thickness) as well as the sensitivity (proportional to the inverse thickness) to the application requirements. Calculations combining the sensor model with the measurement results indicate that for operation in the linear range, about 3% of the LNT material is converted to nitrate.

## 1. Introduction

Tightened emission and safety regulations have increased the demand for sensitive devices to detect reliably even low levels of NO and NO_2_ (NO_x_) over a long measurement period [1–3] (e.g., summarized as <100 ppm NO_x_ in the automotive exhaust and 0.5–5 ppm NO_2_ in the interior by [1]). In the field of automotive or industrial exhausts or of air quality management, the interest is on the accurate determination of mean values (e.g., 1-h value for air quality monitoring [4]) or total amounts (e.g., cumulated vehicle emissions in g/km for on-board diagnostics [5,6]) rather than on the curve of the actual instantaneous concentration over time. However, most today's gas sensors measure time-continuously the actual analyte concentration [1]. Cumulated amount mean values are obtained by mathematical averaging (integration). Due to inaccuracies at low analyte levels, long sensor response times and recovery times, as well as due to drifts in the zero point level (baseline), these sensors are subject to errors in the determination of the accumulated analyte levels [2,3,7].

Alternatively the accumulating-type (or integrating-type or dosimeter-type) sensor measures directly the total amounts of analyte gases over a time interval. This novel principle is related to passive samplers being used to determine the cumulated analyte level in two steps. There, over a longer period (e.g., a month) analyte molecules from the ambience are collected in a diffusion controlled process on a sorption material, followed by a quantitative analysis with laboratory gas analysis methods [8,9]. Accumulating-type gas sensors presented here are also based on a sensitive layer that collects analyte molecules over a longer period, but in contrast to passive samplers, the analyte level is evaluated instantaneously and time-continuously by electrical means. While chemically sorbing the analyte molecules, the electrical properties of the sensitive layer, e.g., the resistivity, change with the amount of analyte stored. Like in passive samplers, the sorbent material needs to be regenerated periodically as saturation effects occur. The sensor signal of the accumulating NO_x_ sensor correlates directly with the total amount of NO_x_ (being the sum of NO and NO_2_), whereas the curve of the actual concentration can be obtained from the timely derivative of the sensor signal [10]. By collecting analyte molecules from the gas stream, even small levels contribute to the sensor signal, enabling accurate analyte detection over an extended time interval. Another important feature is the fact that errors in the sensor's zero level are minimized since the zero-level is redefined after each regeneration step. Both response times and recovery times of the signal derivative as the measurand for the actual concentration are quite low and in the range of the gas exchange time of the setup (<7 s [10]). The fast sensing characteristics of the accumulating sensor originate from the fact that the change (*i.e.*, the time-based derivative) in the conductivity during NO_x_ exposure correlates with the NO_x_ concentration. This is in strong contrast to common gas sensors, in which the equilibrium conductivity of the sensor signal is of interest. In the following, the effect of the thickness of the NO_x_ storage layer on the NO_x_ accumulating sensing properties is addressed.

## 2. The Accumulating Sensing Concept

The accumulating or integrating-type sensor is intended to detect directly the total amount, *A*, of low levels of analyte by accumulation. Generally, *A* can be calculated according to Equation (1) from the analyte concentration, *c*(*t*), and the flow rate, *V̇*(*t*). Both may vary with time.

(1) $$A=\int c(t)\cdot \dot{V}(t)\text{d}t$$

In [11] two setups of the integrating NO_x_ sensor are presented. Utilizing a special channel-type setup with a large area of sensitive material compared to the small gas volume inside the channel, all analyte molecules become sorbed and the resulting sensor signal reflects the total amount, *A*, even if the flow rate of the gas varies (amount detector). Contrarily, it was investigated in [11] that by exposing the sensitive layer of the planar device to a large gas volume, always a constant fraction of the analyte molecules in the gas stream is stored independently on the gas flow rate in a wide range. The sensor signal correlates then with the timely integral of the concentration, ∫*c*(*t*)d*t*. If the flow rate, *V̇*(*t*), remains constant, *A* is directly proportional to the integral of *c*(*t*), *i.e.*, *A* ∝ ∫*c*(*t*)d*t* and the total amount can be determined properly with the planar setup while the signal derivative reflects the curve of *c*(*t*) [10].

**Figure 1.**
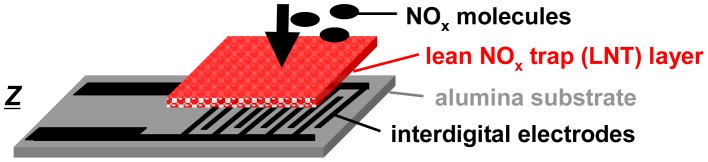
Planar sensor setup of the impedimetric accumulating NO_x_ sensor based on a lean NO_x_ trap layer deposited on interdigital electrodes.

**Figure 2.**
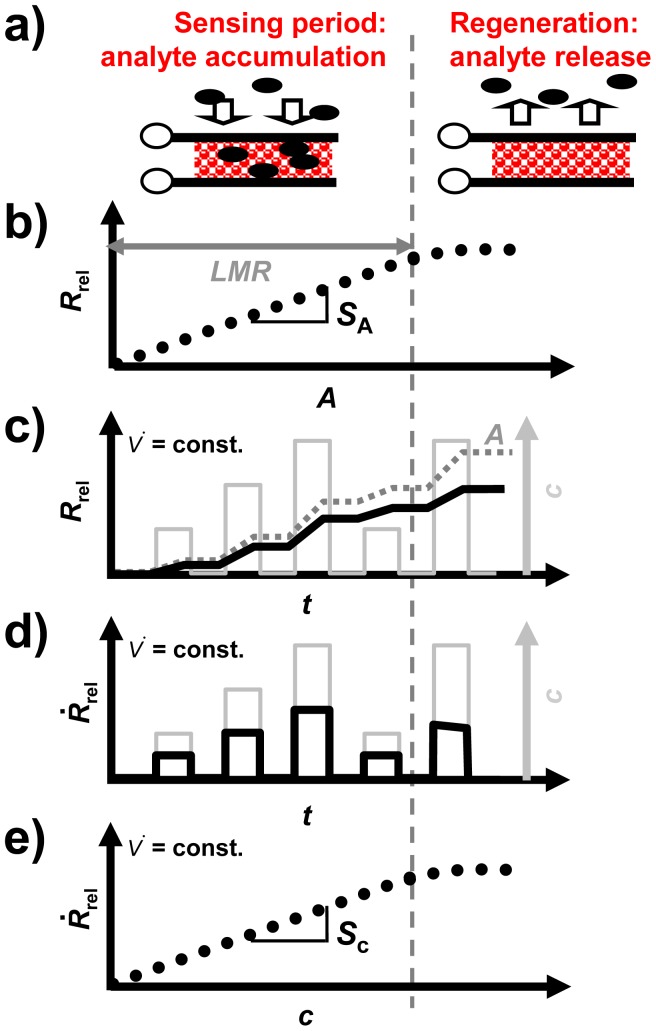
Scheme of the accumulating sensing principle: (**a**) Operation with alternating sensing and regeneration periods; (**b**) characteristic line to determine the total amount: linear correlation between the sensor response and the amount *A* in the linear measurement range, *LMR*; (**c**) sensor signal on the time scale, *R*_rel_ (black full line), increases in the presence of NO_x_; amount *A* (dotted grey line) calculated acc. to Equation (1); (**d**) signal derivative *Ṙ*_rel_ reflects the actual concentration *c*; (**e**) correlation between *Ṙ*_rel_ and *c*.

For the accumulating sensing principle sensitive layers of storage materials can be applied. They are able to sorb analyte molecules (e.g., by chemisorption or by a chemical reaction) and thereby they change their electrical properties. As illustrated in Figure 1, the accumulating NO_x_ sensor in the planar setup (concentration integrator) consists of a lean NO_x_ trap (LNT) layer deposited on an alumina substrate which is equipped with interdigital electrodes (IDEs). It is well known that LNT materials lower their resistivity when transformed from carbonates to nitrates upon NO_x_ storage [12–15]. Since the accessible sorption sites in the storage material are becoming occupied with proceeding sorption, saturation effects limit the accumulating properties and a regeneration of the sorption sites is required to recover the original sorption capacity. Therefore, as illustrated in Figure 2(a), in the operation of accumulating sensors the sensing interval during which the sensitive layer (shaded area) accumulates the analyte molecules (black points) from the gas stream alternates with the regeneration interval. The absolute value of the relative resistance change, *R*_rel_, calculated by Equation (2) with *R*_0_ being the base resistance in the unloaded state, is displayed as the accumulating NO_x_ sensor signal:

(2) $$R_{\text{rel}}=\frac{\left|\Delta R\right|}{R_{0}}=\frac{R_{0}-R}{R_{0}}$$

*R*_rel_ depends on the loading level which, for low loading states, is proportional to the amount of NO_x_ in the gas phase, *A*, as illustrated in Figure 2(b). The sensor signal on the time scale differs from those of conventional gas sensors due to the stepwise NO_x_ accumulation. As shown in Figure 2(c), *R*_rel_ (black line) increases in the presence of NO_x_, whereas it remains constant in the NO_x_ absence (holding ability)—*R*_rel_ is proportional to *A* (dark grey line, dotted). In the case of a constant flow rate, *V̇*(*t*), the timely increase of the sensor signal is proportional to the NO_x_ concentration *c* (light grey line, right axis). This proportionality enables to determine the curve of the instantaneous concentration using the timely derivative, d*R*_rel_/d*t*. In the following, d*R*_rel_/d*t* will be denoted as *Ṙ*_rel_:

(3) $${\dot{R}}_{\text{rel}}=\frac{\text{d}R_{\text{rel}}}{\text{d}t}$$

*Ṙ*_rel_ is illustrated as a function of time in Figure 2(d). As depicted in Figure 2(e), there is a linear correlation between *Ṙ*_rel_ and *c*. Hence, to compare the performance of the accumulating sensor with conventional gas sensors, the characteristics of *Ṙ*_rel_ are appropriate. The sensitivity is commonly defined as the slope of the characteristic line which is the correlation between the sensor signal and the measurand [16]. Hence, for the accumulating type NO_x_ sensor, which is intended to determine *A*, the amount sensitivity, *S*_A_, (Figure 2(b)) can be calculated from the correlation between *R*_rel_ and *A* according to Equation (4) resulting in the unit %/μL. Additionally, in the case of a constant flow rate, the proportionality between *Ṙ*_rel_ and *c*, as shown in Figure 2(e), allows to calculate the concentration sensitivity, *S*_c_, according to Equation (5) which is analogous to the sensitivity known of conventional gas sensors:

(4) $$S_{\text{A}}=\frac{\text{d}R_{\text{rel}}}{\text{d}A}$$

(5) $$S_{\text{c}}=\frac{\text{d}(\text{d}R_{\text{rel}}/\text{d}t)}{\text{d}c}=\frac{\text{d}{\dot{R}}_{\text{rel}}}{\text{d}c}$$

If one transforms Equation (4) using Equation (1), one obtains:

(6) $$S_{\text{A}}=\frac{\text{d}R_{\text{rel}}}{\text{d}A}=\frac{\text{d}R_{\text{rel}}/\text{d}t}{\text{d}A/\text{d}t}=\frac{{\dot{R}}_{\text{rel}}}{\text{d}\left(\int c(t)\cdot \dot{V}(t)\text{d}t\right)/\text{d}t}=\frac{{\dot{R}}_{\text{rel}}}{\dot{V}\cdot c}$$

If *S*_c_ = const, *i.e.*, *Ṙ*_rel_ ∝ c, then d*Ṙ*_rel_/d*c* can be replaced by *Ṙ*_rel_/*c*. Then the relation between *S*_c_ and *S*_A_ yields:

(7) $$S_{\text{A}}=\frac{1}{\dot{V}}S_{c}$$

In other words, the amount-related sensitivity, *S*_A_, and the “classical” sensitivity with respect to the concentration, *S*_c_, are proportional to each other, as long as the gas flow remains constant. As shown in Figure 2(b,e), as soon as saturation effects occur and the linear measurement range, *LMR*, is exceeded, *S*_A_ and *S*_c_ decrease, and the slope of the signal no longer reflects the concentration (Figure 2(b–e))—the accumulating sensor demands regeneration.

LNT materials are known from automotive NO_x_ storage and reduction catalysts to reduce the NO_x_ emissions in the exhaust [17–19]. Since NO_x_ molecules can be stored in a lean gas atmosphere, whereas they are released and reduced in rich gas compositions, the engine operation cycles between long lean and short rich intervals to ensure low emissions [5,17–19]. LNTs usually consist of alkaline (earth-) oxides or carbonates (e.g., BaCO_3_ or K_2_CO_3_) as storage components, finely dispersed precious metal particles to catalyze oxidation and reduction reactions, and support oxides like Al_2_O_3_ to provide high surface areas for the catalytic processes [19,20]. The storage mechanism is based on the conversion of alkaline (earth-) carbonates MCO_3_ or oxides to nitrates M(NO_3_)_2_ upon NO_2_ exposure according to Equation (8). NO needs to be oxidized to NO_2_ on the catalytic active particles prior to the nitrate formation according to Equation (9) [17,18,20]:

(8) $$MCO_{3}+2NO_{2}+\frac{1}{2}O_{2}↔M{\left(NO_{3}\right)}_{2}+CO_{2}$$

(9) $$NO+\frac{1}{2}O_{2}↔NO_{2}$$

We recently demonstrated the integrating or accumulating NO_x_ sensing principle under various gas conditions (base gas composition, temperature) [10,21,22]. Additionally, it was found that the sensitivity to NO is the same as that to NO_2_, thus allowing for total NO_x_ detection, and that the sensor is suitable for long-term detection of low levels of NO_x_ [10]. O_2_ and CO_2_ concentration variations were found to be negligible in a wide range in lean gas containing O_2_, CO_2_ and H_2_O [10,21].

In order to understand further how NO_x_ storage occurs in the catalyst material, the influence of the thickness of the sensitive layer on the performance of the accumulating NO_x_ sensor is the focus of this study. This is motivated by the idea that the number of accessible storage sites and hence the fraction of sites occupied by NO_x_ upon NO_x_ exposure should depend on the thickness of the LNT coating if the LNT coated area remains the same. The obtained results may even be of interest for LNT catalyst research and may help to elucidate more details about the storage reactions.

## 3. Expected Influence of the Thickness of the Sensitive Layer—Some Pre-Considerations

**Figure 3.**
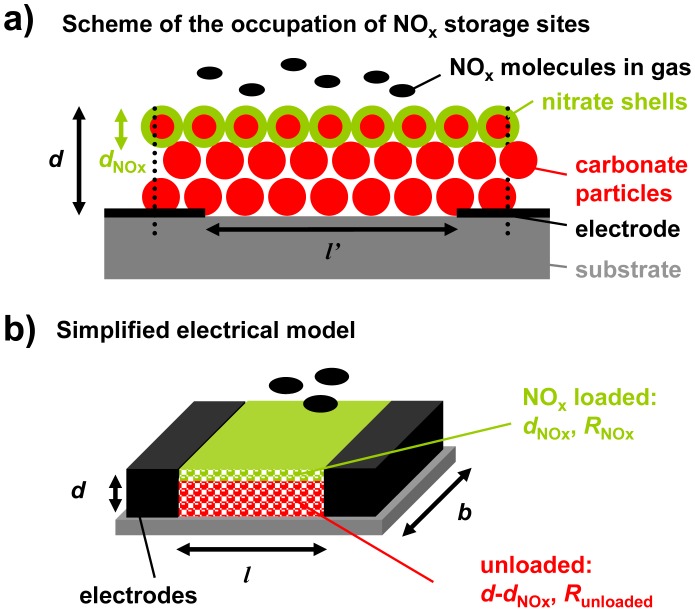
Model of the influence of the thickness of the sensitive layer on the electrical properties: (**a**) Mechanism of the storage of NO_x_ as nitrate shells on carbonate cores at the surface of the NO_x_ storage layer; (**b**) simplified electrical model consisting of a parallel circuit of the NO_x_ loaded material and the remaining unloaded layer.

The storage capacity and hence the number of accessible storage sites control the analyte accumulation properties of LNT catalysts. Hence, it is expected that the thickness of the carbonate layer affects the accumulating sensing properties. As described in Figure 3, a simplified model of the configuration containing the storage material (red) and the interdigital electrodes (black) was developed for a rough estimation of this influence.

From catalyst research it has been known that NO_x_ storage occurs mainly at the surface of the LNT material that is in contact with the gas phase, resulting in less than 40% utilization of the available storage sites upon saturation [23–25]. This means that even in the highly loaded state, only a fraction of the storage sites are involved in the storage process. Additionally, nitrate formation is accompanied by a shrinking of the pore structure since the nitrates have a higher molar volume than the corresponding carbonates [26]. In the case of K_2_CO_3_, the volume theoretically increases by almost 70% upon storing NO_x_. The decreased diameter of the pores lowers the diffusion of the NO_x_ molecules into the carbonate particles and the NO_x_ loaded zones can be modeled as dense nitrate shells at the surface of the LNT particles (shrinking core type model [26,27]). Since the accumulating NO_x_ sensor is exposed to small NO_x_ concentrations and is only operated in the low loading state, nitrate shells are expected to form mainly at the upper surface area of the LNT layer, which is in close contact to the analyte gas phase. Hence, the occupation of NO_x_ storage sites in the sensitive layer by NO_x_ molecules can be illustrated as shown in Figure 3(a). Thereby, the thickness of the NO_x_ loaded area, *d*_NOx_, increases with progressive NO_x_ exposure. If the distance between the electrodes, *l*′, is much larger than the film thickness, *d*, the electrical field lines between the electrode fingers are parallel and homogenously distributed. Almost the entire flux is inside the material [28]. Hence, in a simplified model of the sensor setup, the LNT layer can be described as a resistive material in between two parallel electrodes with the distance *l* (Figure 3(b)). The term *l* is related to the distance of the planar electrodes of the IDEs, *l′*. The relation between *l* and *l′* can be calculated which has even been experimentally proven in [29].

Applying this simplified model of Figure 3(b) for the case of the regenerated state (*d*_NOx_ = 0), the base resistance of the accumulating NO_x_ sensor in the unloaded state, *R*_0_, can be calculated from the geometry and the resistivity of the carbonate material, *ρ*_0_, by Equation (10). Therefore, it is expected that *R*_0_ correlates with the inverse thickness, 1/*d*:

(10) $$R_{0}=\rho_{0}\frac{l}{bd}\propto \frac{1}{d}$$

Upon exposure to NO_x_, it is assumed that surface nitrate is formed. Hence, the corresponding simplified model in the partly loaded state contains a thin nitrate film with the resistance *R*_NOx_ and the thickness *d*_NOx_ on top of the remaining unloaded material with the resistance *R*_unloaded_, as shown in Figure 3(b). The resulting resistance of the sensitive layer, *R*, can be calculated as a parallel circuit of both fractions (*R*_NOx_‖*R*_unloaded_). The sensor signal *R*_rel_ can then be calculated from the sensor geometry, the resistivity of the sensitive material in the unloaded state, *ρ*_0_, and the resistivity of the NO_x_ loaded material, *ρ*_NOx_, according to Equation (11):

(11) $$R_{\text{rel}}=\frac{\left|\Delta R\right|}{R_{0}}=\frac{1}{\frac{d}{d_{\text{NOx}}(\rho_{0}/\rho_{\text{NOx}}-1)}+1}$$

*d*_NOx_ can be assumed to be very small compared to the thickness of the sensitive layer, *d*, since only the lightly loaded state is considered. If it is further considered that the resistivity decreases by at least one order of magnitude in the presence of NO_x_, Equation (11) can be simplified to Equation (12) meaning that *R*_rel_ is proportional to 1/*d*:

(12) $$R_{\text{rel}}\approx \frac{d_{\text{NOx}}\cdot (\rho_{0}/\rho_{\text{NOx}}-1)}{d}\propto \frac{1}{d}$$

Since *A* is independent of *d* and the same amount of NO_x_ is expected to result in the same thickness of the formed NO_x_ loaded layer, Equations (4) and (12) lead to Equation (13) and the amount-sensitivity, *S*_A_, should correlate with 1/*d* as well:

(13) $$S_{\text{A}}=\frac{\text{d}R_{\text{rel}}}{\text{d}A}\approx \frac{\rho_{0}/\rho_{\text{NOx}}-1}{d}\cdot \frac{\text{d}(d_{\text{NOx}})}{\text{d}A}\propto \frac{1}{d}$$

As the resistivity decreases by at least one order upon saturation in NO_x_, (*ρ*_0_/*ρ*_NOx_−1) ≥ 10 and Equation (13) can be simplified to Equation (14):

(14) $$S_{\text{A}}=\frac{\text{d}R_{\text{rel}}}{\text{d}A}\approx \frac{\rho_{0}}{\rho_{\text{NOx}}}\cdot \frac{1}{d}\cdot \frac{\text{d}(d_{\text{NOx}})}{\text{d}A}\propto \frac{1}{d}$$

Since *S*_A_ ∝ *S*_c_ for constant gas flows, even the classical concentration-related sensitivity, *S*_c_, is expected to depend on 1/*d* and to increase the thinner the sensing layers are.

This simplified model points out that the thickness of the sensitive layer might be an effective tool to vary the sensing properties (especially the sensitivity) of the accumulating NO_x_ sensor. In the following study, the model was validated by exposing samples with various thicknesses to NO_x_ containing gas flows and monitoring the sensing performance.

## 4. Experimental

Samples with coatings of different thicknesses were prepared and exposed to various gas compositions containing NO, NO_2_ or total NO_x_. As illustrated in Figure 1, the accumulating NO_x_ sensor consists of an LNT layer (potassium-based LNT material provided by Johnson Matthey, composition details given in [30]) deposited on platinum interdigital electrodes (IDEs) with an area of 5 × 6 mm^2^ and an electrode width and spacing of 100 μm on an alumina substrate with a purity of 96%. After drying and milling the catalyst powder, a screen-printable paste was made by adding organic additives (KD 2721, Zschimmer & Schwarz). To obtain samples with sensitive layers in various thicknesses, the IDE area was screen-printed multiple times with the LNT-paste with intermediate drying periods. After firing at 650 °C to remove organic additives, the sensing properties of the samples were analyzed at 380 °C in a sensor test bench. Thereby, lean measurements periods and rich desorption periods were periodically applied. The gas flow was kept constant at *V̇*(*t*) = 2 L/min. The base lean gas consisted of 10% O_2_, 5% CO_2_, and 50% N_2_ humidified (by a water bubbler at room temperature) in N_2_, whereas the rich gas for regeneration contained 1.5% H_2_, 5% CO_2_, and 50% N_2_ humidified in N_2_. Different NO_x_ gas compositions were admixed. The NO and NO_2_ concentrations were monitored by a chemiluminescence detector downstream of the sensor sample. The complex impedances of the sensitive devices were measured in the frequency range from 0.1 Hz to 20 MHz. The electrical characteristics of the bulk material can be described by an *R*‖*C* parallel equivalent circuit. In time-continuous measurements, *R* was calculated from the impedance taken at 1 kHz applying the *R*‖*C* model. The thicknesses of the sensitive layers were estimated using SEM micrographs.

## 5. Results and Discussion

### 5.1. Thickness Determination from SEM Analysis

**Figure 4.**
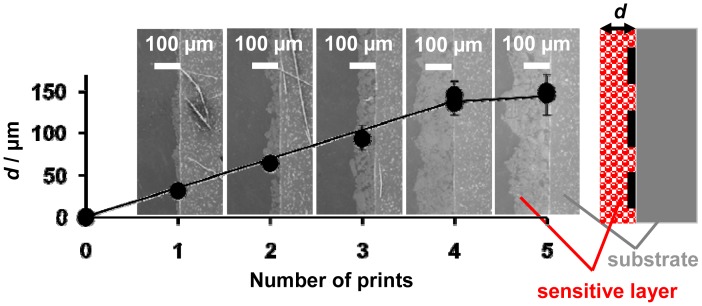
SEM analysis to estimate the thickness of the sensitive layers as a function of the number of prints.

SEM images from the cross sections of the sensor samples printed multiple times with the LNT paste and an illustration for clarification reasons are shown in Figure 4. The microstructure of the sensitive layers is dominated by loose grains of different diameters. The thickness increased with each printing step from about 30 μm (printed only once) to 150 μm (printed five times). The samples printed four and five times had almost the same thickness. This might be due to a densification of the LNT material with successive printing as it is well known when printing porous films or due to a erroneous thickness determination as a result of the increased roughness. In general, the surface is very rough, with an increasing roughness in the case of thicker coatings lowering the accuracy of the thickness evaluation.

### 5.2. Base Resistance as a Function of the Thickness

**Figure 5.**
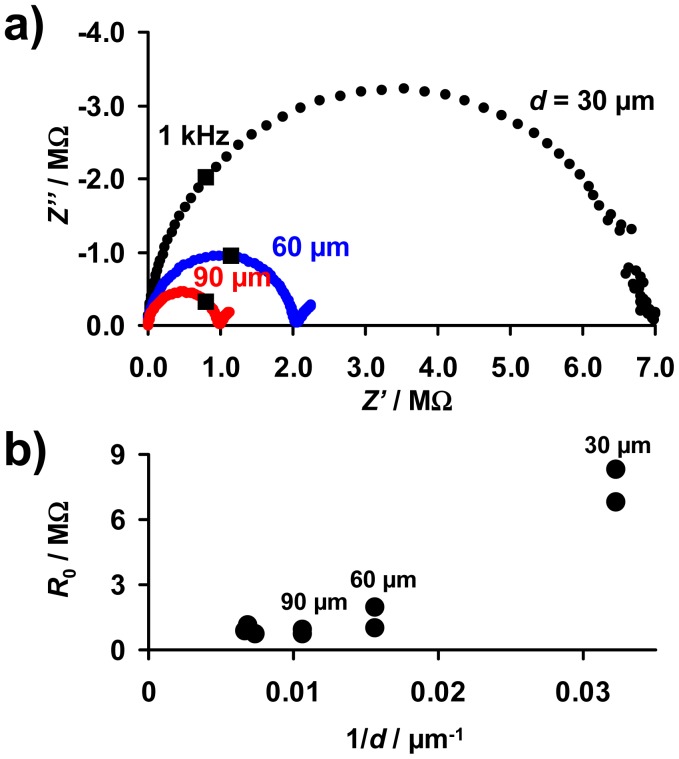
Effect of the thickness of the sensitive layer on the electrical properties in the unloaded state: (**a**) Complex impedance plots of samples with 30 μm, 60 μm, and 90 μm (data points at 1 kHz are marked); (**b**) base resistance, *R*_0_, as a function of the inverse thickness, 1/*d*.

As described above, the base resistance of the sensitive layer in the unloaded state, *R*_0_, is expected to be proportional to 1/*d* (Equation (10)). For an electrical characterization of the sensitive layer, complex impedance plots of the samples with sensitive LNT layers of 30 to 150 μm were taken. The electrical properties of all investigated sensor samples between 10 Hz and 20 MHz can be described by an *R*‖*C* equivalent circuit. Figure 5(a) shows exemplarily Nyquist plots of samples with layers of 30, 60, and 90 μm. The data points measured at 1 kHz are marked. Fitting the spectra by an *R*‖*C* model, the values of *R*_0_ were obtained and are plotted as a function of 1/*d* in Figure 5(b). *R*_0_ increases with decreasing thickness, although no exact 1/*d*-dependency occurs. The resistances of the samples with the thinnest layers (30 μm) are especially high, resulting in deviations from the expected 1/*d*-behavior (Equation (10)). This may be caused by the loose packing of the catalyst particles in the coating and by inaccuracies in the thickness estimation due to the film unevenness. Furthermore, the simple model depicted in Figure 3(a) that leads to Equation (10) is only valid if the layers are by far thinner than the distance between the IDE fingers (*d* ≪ *l′*). This was confirmed in [28] by modeling the electrical flux lines in a system of a highly resistive substrate, metallic IDEs, and a resistive sensitive layer covering the IDEs. Since the distance between the electrodes of the applied IDE samples is 100 μm, it is assumed that in samples with coating of 100 μm and above there are less flux lines in the outer LNT material. Therefore, the outer parts of the LNT layer do not or only slightly contribute to the overall resistance. As a result, these thicker films may no longer exhibit the *R*_0_ ∝ 1/*d* behavior.

### 5.3. Direct Accumulative Amount Detection

**Figure 6.**
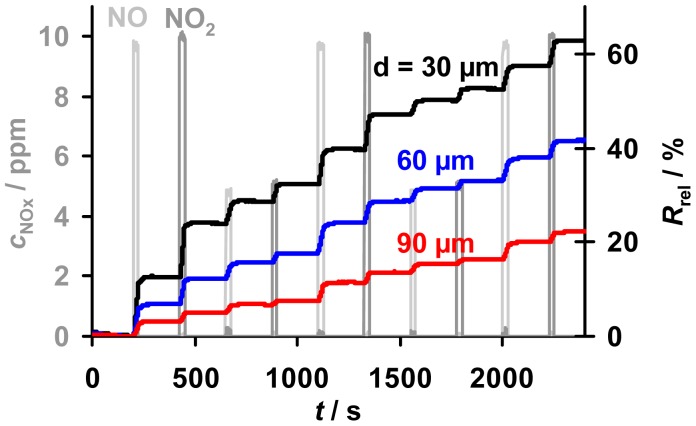
Accumulating sensor responses, *R*_rel_, of the samples with various thicknesses increasing stepwise during cyclic exposure to 5 or 10 ppm NO (light grey line) or NO_2_ (dark grey line) for 25 s each alternating with 0 ppm NO_x_ for 200 s.

A cyclic test program to investigate the accumulating sensing properties of the samples with various thicknesses in low levels of NO_x_ (here 10 ppm and less), especially the holding capability in the absence of NO_x_, is given in Figure 6. Also shown is a comparison of the sensor responses, *R*_rel_, towards NO and NO_2_. The samples with layers from 30 to 90 μm were exposed to alternating NO and NO_2_ steps of 25 s with concentrations of 5 and 10 ppm interrupted by NO_x_ pauses of 200 s. Due to the lower resistivity of the nitrate compared to the carbonate form of the storage material, the resistance decreased during NO_x_ loading, yielding an increase in *R*_rel_ [10,13]. For all samples in Figure 6, *R*_rel_ increases linearly at constant NO_x_ concentration, with an almost equal response to NO and NO_2_, while *R*_rel_ remains constant in 0 ppm NO_x_. It is evident that the sensors are less sensitive to NO_x_ the thicker the storage layers are, albeit the samples are exposed to the same NO_x_ profile. The constancy of *R*_rel_ during the 200 s intervals without NO_x_ reveals that all sensitive layers are able to keep the stepwise accumulated NO_x_ molecules - even in the absence of NO_x_. This indicates that the storage capacities of the samples with various thicknesses are not nearing saturation and that the formed nitrates are highly stable in the applied conditions enabling accumulating NO_x_ sensing. Hence, all applied samples with various thicknesses provide accumulating NO_x_ sensing properties.

A more detailed analysis of the dependency of the sensitivity, the linear measurement range and the sensor response time on the thickness of the sensitive layer is given in the next sections.

### 5.4. Concentration Detection by the Signal Derivative

**Figure 7.**
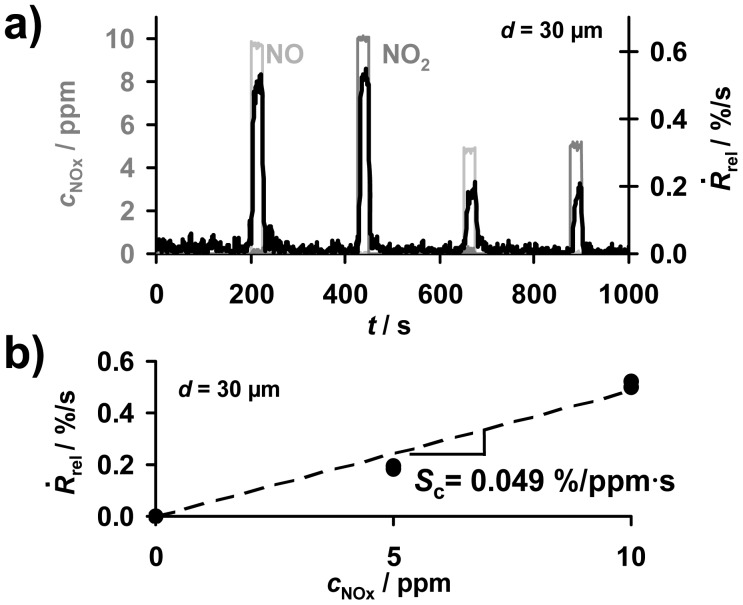
Signal derivative, *Ṙ*_rel_, of the 30 μm sample during cyclic exposure to NO and NO_2_: (**a**) *Ṙ*_rel_ of the 30 μm sample corresponding with the actual NO_x_ concentration, *c*_NOx_; (**b**) resulting correlation between *Ṙ*_rel_ and *c*_NOx_.

The progressive accumulation of NO_x_ molecules in the sensitive layer in the presence of NO_x_ enables the direct detection of the total NO_x_ amount. As sketched in Figure 2, in the case of a constant flow rate, the time-based signal derivative, *Ṙ*_rel_ according to Equation (3), of the ideal accumulating sensor in the low loaded state can be applied to obtain information about the actual NO_x_ concentration, *c*_NOx_. To compare accumulating-type sensors with conventional gas sensors, one has to analyze the concentration sensitivity *S*_c_, *i.e.*, the derivative *S_c_* = d*Ṙ*_rel_/*d_c_* according to Equation (5). Exemplarily, the corresponding data of the signal derivative *Ṙ*_rel_ of the sample coated with a 30 μm storage layer are plotted in Figure 7(a). During the first four NO_x_ periods, *Ṙ*_rel_ reflects the curve of *c*_NOx_ being the sum of *c*_NO_ and *c*_NO2_ allowing for determining the actual NO_x_ concentration. The corresponding characteristic line for the concentration detection correlating *Ṙ*_rel_ and *c*_NOx_ is given in Figure 7(b). *Ṙ*_rel_ increases with an increased NO_x_ concentration in the gas. Hence, the NO_x_ sensitivity, *S*_c_, of the 30 μm sample can be calculated from Equation (5) and was found to be 0.049%/ppm·s. This means that the resistance decreases by 0.049%/s upon exposure to 1 ppm NO_x_. The analysis of the sensor response times for the concentration detection by *Ṙ*_rel_ in dependency of the thickness of the sensitive layer will be discussed in detail in Sections 5 and 6. From *Ṙ*_rel_ in Figure 7, one obtains a sensor response and recovery time of about 5 to 8 s, which is in the range of the gas exchange time of the setup. Figure 7 demonstrates that besides detecting directly the total NO_x_ amount, the signal derivative of the accumulating NO_x_ sensor allows NO_x_ concentration monitoring with a high sensitivity and a fast sensor response.

### 5.5. The Effect of the Thickness on the Sensitivity

**Figure 8.**
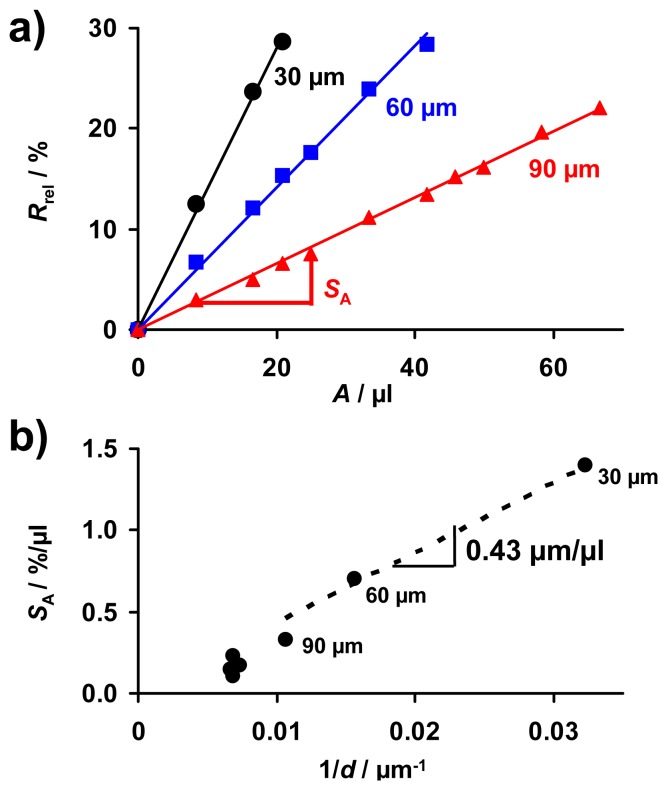
Layer thickness influence on the sensitivity: (**a**) Comparison of the characteristic lines for different coating thicknesses; (**b**) the resulting values of the amount-sensitivity, *S*_A_, as a function of 1/*d*.

According to the simplified model that is based on the assumption that a thin nitrate layer forms at the surface of the sensitive storage layer upon NO_x_ exposure, as shown in Figure 3, and the resulting Equation (13), both sensitivities, *S*_A_ and *S*_c_, should increase with 1/*d*. The characteristic lines, correlating *R*_rel_ with the total NO_x_ amount, *A*, extracted from the cyclic measurement data presented in Figure 6 are shown in Figure 8(a). For each sample, the data points of *R*_rel_ measured at the end of each NO_x_ step form straight lines through the origin up to a sensor response of about 30%, independent of the NO_x_ concentration or the type of NO_x_ (NO or NO_2_). Comparing the characteristic lines for the amount detection of the samples with different thicknesses in Figure 8(a), it becomes clear that *S*_A_ decreases when the sensitive layer gets thicker. To evaluate the thickness effect in detail, *S*_A_ is plotted as a function of 1/*d* in Figure 8(b). *S*_A_ increases linearly with 1/*d* with small deviations in the case of layers being 90 μm or thicker (1/*d* ≤ 0.011 μm^−1^).

From the concentration related sensitivity *S*_c_ ≈ 0.049%/ppm·s of the 30 μm sample determined from Figure 7(b) and the applied gas flow of *V̇* = 2 L/min = 0.033 L/s, the sensitivity S_A_ can be calculated. Applying the relationship between *S*_A_ and *S*_c_ described by Equation (7) one obtains *S*_A_ ≈ 1.48%/μL, which agrees with the value shown in Figure 8(b) for the 30 μm sample. This good agreement verifies that validity of the correlation between *S*_A_ and *S*_c_ (Equation (7)).

These results verify the simplified model as illustrated in Figure 3 and demonstrate that the thickness of the accumulating NO_x_ storage layer can be used as an effective tool to adapt the sensor sensitivity to the application requirements.

According to Equation (14) a linear correlation between *S*_A_ and 1/*d* with a proportionality factor of *ρ*_0_/*ρ*_NOx_·d(*d*_NOx_)/d*A* can be expected from the simplified model. Hence, applying the slope of the correlation in Figure 8(b), d*S*/d(1/*d*), the thickness of the formed nitrate layer, *d*_NOx_, dependent on the exposed amount of NO_x_ can be estimated. Since the resistivity of the samples was found to decrease by at least one order upon saturation in NO_x_ (result not shown here), *ρ*_0_/*ρ*_NOx_ ≈ 10 is assumed as a minimal value. With the slope d*S*/d(1/*d*) being 0.43 μm/μL (dotted line in Figure 8(b) for samples <100 μm), *d*_NOx_ increases by less than about 43 nm per μL NO_x_ in the gas phase.

### 5.6. Variations of the Linear Measurement Range

**Figure 9.**
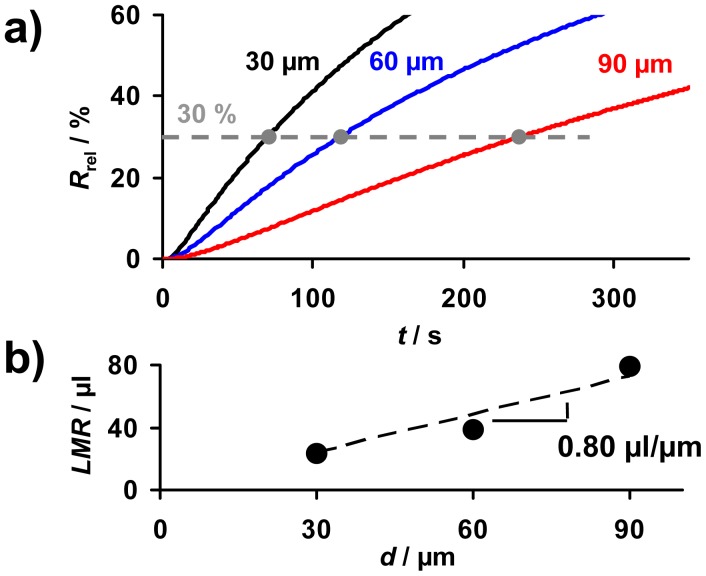
The effect of the thickness on the linear measurement range, *LMR*: (**a**) *R*_rel_ increases linearly in the presence of 5 ppm NO and 5 ppm NO_2_ up to about 30%, (**b**) *LMR* as a function of *d*.

As sketched in Figure 2, the linear measurement range, *LMR*, is defined as the amount of NO_x_ that can be detected when a linear correlation between *R*_rel_ and *A* exists. The *LMR* ends when saturation effects occur and the sensitivities *S*_A_ and *S*_c_ decrease. Therefore, besides the sensitivity and the sensor response time, the *LMR* is an important feature of the accumulating-type sensor. The effect of the sensitive layer thickness on the *LMR* is addressed in Figure 9. In the presence of 10 ppm NO_x_ (consisting of 5 ppm NO and 5 ppm NO_2_) in a lean gas mixture, *R*_rel_ of all samples increases continuously with time (Figure 9(a)). In accordance to Equation (14), the sensitivity is higher in the case of thinner sensitive storage layers. From Figure 9(a) it seems that, independent on the sensitive layer thickness, the *LMR* ends at about *R*_rel_ = 30%. This results in an increase of the *LMR*-amount with the layer thickness as shown in Figure 9(b). The slope in Figure 9(b) indicates that 0.80 μL NO_x_ can be detected linearly per μm LNT layer deposited on the sensor.

In Section 5.5, it was calculated that the thickness of the nitrate fraction of the sensitive layer increases by about 43 nm/μL. This value can be combined with the dependency of the *LMR* on *d* of 0.80 μL/μm from Figure 9(b). One obtains that if reaching the end of the linear measurement range upon NO_x_ exposure 43 nm/μL·0.80 μL/μm = 34 nm nitrate is formed per μm LNT material. In other words, independent of the LNT thickness only about 3% of the sensitive material is converted to nitrate in the case of NO_x_ storage in the linear measurement range. This estimated nitrate fraction of the sensitive layer is much less than the values reported for the storage sites utilization of LNT catalysts of maximal 20 to 40% upon saturation [23–25]. This difference indicates that the end of the linear measurement range of the accumulating NO_x_ sensor might be limited rather by the non-linear relation between resistivity change and NO_x_ loading than by the storage capacity of the LNT material.

### 5.7. Evaluation of the Sensor Response Time

**Figure 10.**
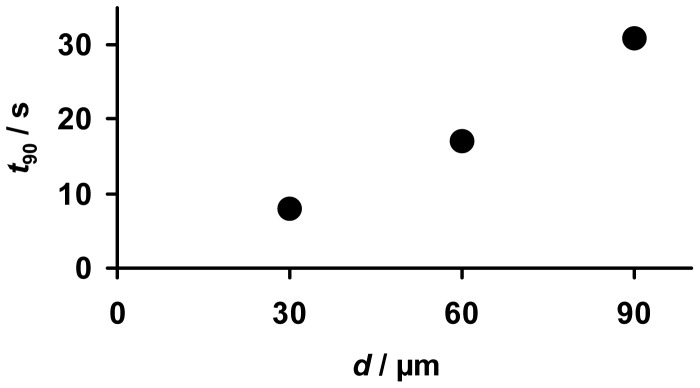
Analysis of the sensor response time, *t*_90_, of *Ṙ*_rel_ in 10 ppm NO_x_ as a function of the thickness *d*.

With the accumulating NO_x_ sensor the amount of NO_x_ is detected by looking on the changes in the electrical properties and not on the equilibrium values like with conventional gas sensors. Hence, no sensor response time of *R*_rel_ can be defined in the classical way. Instead one has to apply the timely derivative, *Ṙ*_rel_, which is a function of the concentration and therefore corresponds to the sensor signal of conventional gas sensors. From the measured data of the samples in 10 ppm NO_x_, the sensor response time of the slope *Ṙ*_rel_ was analyzed. Therefore, the time to reach 90% of the maximum value of *Ṙ*_rel_, *t*_90_, is compared for the samples with different coating thicknesses. Figure 10 shows that *t*_90_ increases with *d*. While *t*_90_ of the sample with a 30 μm storage layer is about 7 s, it is in the range of 30 s in the case of the 90 μm sample. In the case of very thin coatings the sensor response is limited by the gas exchange of the test bench, which is in the range of 7 s. For very thick coatings (*d* ≈ *l*), NO_x_ storage occurs in a region far away from the electrodes. Due to the increasingly weaker electrical field lines, NO_x_ uptake in this region of the LNT layer is most probably not reflected properly by *R*_rel_ and *Ṙ*_rel_. Additionally, it is expected that the accessibility of the storage sites is dependent on the LNT thickness as with progressive NO_x_ loading upcoming NO_x_ molecules need to diffuse into the catalyst material to reach unoccupied storage sites [26,27,31]. The analysis of the sensitivity and the sensor response time in Figures 8 and 10 clarifies that for a highly sensitive and fast low level detection of NO_x_, accumulating NO_x_ sensors with a thin sensitive layer are preferable.

## 6. Conclusions

The intent of this study was to investigate the influence of the sensitive layer thickness on the sensing properties of the accumulating NO_x_ sensor. In several NO_x_ loading experiments it was demonstrated that the general accumulating NO_x_ amount sensing properties seem not to be affected by the thickness of the sensitive layer in the studied range (*i.e.*, the increase of the sensor signal in the presence of NO_x_ due to NO_x_ accumulation, the correlation between the slope and the NO_x_ concentration, and the constancy of the sensor signal in the NO_x_ absence due to the strength of sorption). The linearity of the sensor signal, *R*_rel_, with the total NO_x_ amount enables the detection of the actual NO_x_ concentration by *Ṙ*_rel_ with all applied samples.

**Figure 11.**
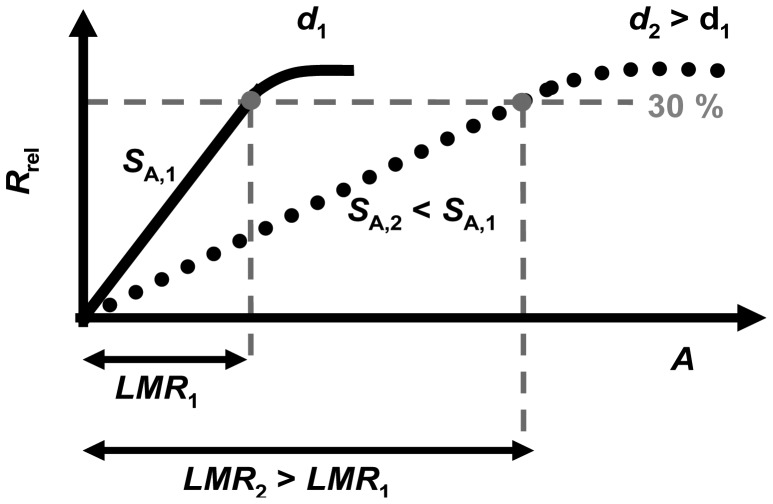
Illustration of the effect of the thickness of the sensitive layer (*d*_1_ < *d*_2_) on the sensitivity, *S*_A_, and the linear measurement range, *LMR*, with the end of the linear measurement range being at 30% signal change.

However, like the base resistance, the sensitivity to NO_x_ is inversely proportional to the film thickness *d*. This agrees with a simple model concerning nitrate formation at the surface of the sensitive layer. It was demonstrated that NO_x_ can be detected linearly until the sensor resistances reaches about 30%. This limit was found to be independent on the thickness of the sensitive layer. This controversial effect of the sensitive layer thickness on the sensitivity, *S*_A_ (and also on *S*_c_), and on the linear measurement range, *LMR*, is illustrated in Figure 11 for two samples with two different thicknesses, *d*_2_ (dark grey line) being higher than *d*_1_ (light grey line). While *S*_1_ is higher than *S*_2_, the resulting *LMR*_2_ is larger than *LMR*_1_. More particularly, *LMR* increases with *d* allowing for a measurement range adaption depending on the requirements of the application conditions. However, there is a trade-off between a large linear measurement range and a high sensitivity.

The presented measurement results also point out that the timely sensor response characteristic depends on the thickness of the storage material. In the case of very thin layers (30 μm) the sensor response time corresponds to the gas exchange time of the gas flow stand, whereas the sensor signal becomes slower as the thickness increases. An estimation based on the presented simplified model of the sensor setup reveals that, independently on the thickness of the LNT material only a small fraction of the sensitive layer—probably about 3%—is involved in the NO_x_ storage process as the accumulating sensor is operated in the linear measurement range.
